# NAT10 promotes the tumorigenesis and progression of laryngeal squamous cell carcinoma through ac4C modification of FOXM1 mRNA

**DOI:** 10.1080/15384047.2023.2274143

**Published:** 2023-11-10

**Authors:** Zengpei Li, Dajun Li, Tianbin Yang, Chen Yao

**Affiliations:** Department of Otolaryngology, Nanyang First People’s Hospital, Nanyang, Henan, China

**Keywords:** NAT10, FOXM1, ac4C, LSCC, proliferation, migration

## Abstract

Laryngeal squamous cell carcinoma (LSCC), is a prevalent malignant tumor, belongs to the category of head and neck tumors. N-acetyltransferase 10 (NAT10) can alter mRNA stability through N4- acetylcytidine (ac4C) modification. This study aimed to make an investigation into the role of NAT10-mediated ac4C modification in the malignant processes of LSCC cells. The NAT10 expression in LSCC tissues and cells was detected RT-qPCR and western blot. The ac4C dot blot was performed to detect ac4C level. Besides, the cell viability, migration, and invasion abilities were detected by CCK-8 and transwell assays. AcRIP-qPCR was performed to measure the abundance of ac4C on FOXM1 mRNA. RIP and Luciferase reporter assays were performed to demonstrate the interaction between NAT10 and FOXM1. Finally, the xenograft model was established to explore the role of NAT10 in vivo. NAT1 levels were significantly increased in the LSCC tissues and cells. Knockdown of NAT10 could significantly suppress the proliferation, migration, and invasion of LSCC cells. Additionally, NAT10 recognized the ac4C-modified sites in the 3’-untranslated regions (3’ UTR) of forkhead box M1 (FOXM1) to enhance the ability of FOXM1 mRNA. Furthermore, FOXM1 overexpression reversed the suppressing effects of NAT10 knockdown on the proliferation, migration, and invasion of LSCC cells, according to the results of rescue assays. Finally, results of animal experiments showed that NAT10 promoted in vivo tumorigenesis of LSCC cells through upregulating FOXM1. Our current study demonstrated that NAT10-mediated ac4C modification of FOXM1 mRNA promoted the malignant processes of LSCC cells.

## Introduction

Laryngeal squamous cell carcinoma (LSCC), a highly prevalent sub-type of laryngeal cancer, has relatively high mortality.^1^ Although advancements have been made in therapies such as surgical resection, chemotherapy, and combined therapies, the prognosis of LSCC patients remains unimproved significantly due to late diagnosis and distant metastasis.^2,3^ As such, it is crucial to explore novel biomarkers and targets for the prognosis and treatment of LSCC patients.

N-acetyltransferase 10 (NAT10), is a lysine acetyltransferase (KAT) that acetylates multiple RNAs, which belongs to the general control non-repressible 5 (GCN5)-related N-acetyltransferase (GNAT) family.^4–7^ As reported, NAT10 can affect cellular physiology by acting as an acetylation regulator to modulate RNA transcription and stability.^8–10^ More importantly, NAT10 is related to the progression of various malignant tumors, such as hepatocellular carcinoma,^11^ pancreatic cancer,^12^ and breast cancer.^13^ However, the role of NAT10 in LSCC has not been reported in detail.

N4-acetylcytidine (ac4C) is a conservative chemical modification of tRNA [4], rRNA,^5^ and mRNA.^6^ ac4C catalyzed by NAT10 on tRNAs or rRNA can efficiently increase the fidelity or accuracy of protein translation.^14,15^ It has been found that ac4C is widely present on mRNA wobble sites, which can help to maintain mRNA stability and improve translation efficiency.^16^ To date, whether NAT10-mediated ac4C modification can regulate LSCC progression remains unclear. This study aims to reveal the role of NAT10-mediated ac4C modification in the malignant processes of LSCC cells.

Forkhead box M1 (FOXM1) is a known transcription factor^17,18^ and a positive effector of the Wnt pathway.^19^ Elevated FOXM1 expression is observed in various human malignant tumors, and silencing of FOXM1 can efficiently suppress the malignant phenotype of cancer cells.^20,21^ Importantly, multiple studies have demonstrated the critical role of FOXM1 in LSCC as well.^22–24^ Therefore, uncovering novel regulating mechanisms of FOXM1 is conducive to providing new insights into the therapeutic strategies against LSCC. To our knowledge, whether NAT10 can regulate FOXM1 expression through ac4C modification to exert functions in LSCC remains to be investigated.

To summarize, this study focused on investigating the effects of NAT10-mediated ac4C of FOXM1 on LSCC growth and metastasis.

## Materials and methods

### Tissue collection

Nineteen pairs of LSCC tissue samples and adjoining normal tissues were obtained from 19 LSCC patients through surgical resection, under approval from Nanyang First People’s Hospital. The written informed consent was signed by each participant before sample collection. No patient received any treatment before admission. All tissues were frozen in liquid nitrogen as soon as extraction and stored at − 80°C until further experiments.

## Cell culture

Human LSCC cell lines (TU686 and AMC‐HN‐8) and the healthy bronchial epithelial cell line (HBEC2) were obtained from Cell Bank of the Chinese Academy of Sciences (Shanghai, China) and were kept at 37°C in a humidified atmosphere with 5% CO_2_. The Dulbecco’s modified Eagle’s medium (DMEM, HyClone, South Logan, UT, USA) containing 10% fetal bovine serum (FBS, Invitrogen, CA, USA) was used for cell culture.

## Cell transfection

The short hairpin RNAs targeting ERCC6L (shERCC6), KIF4A (shKIF4A), and scramble shRNA (negative control, shCtrl) were synthesized by GenePharma (Shanghai, China). For FOXM1 overexpression, the pcDNA3.1 vector was sub-cloned with the whole sequence of FOXM1 to construct the FOXM1 expression vector; meanwhile, the empty vector was taken as negative control (named Vector). Transfection was processed using Lipofectamine 2000 (Invitrogen), as instructed by the manufacturer’s protocol.

## Reverse transcription and real-time quantitative polymerase chain reaction (RT-qPCR)

Total RNA extraction was completed using Trizol reagent (Invitrogen). cDNA was obtained by reverse transcription of RNA by using the PrimeScript RT Reagent Kit (TaKaRa, Tokyo, Japan). The reaction system of qPCR was configured using SYBR Green PCR Master Mix (Applied Biosystems, Foster City, CA, USA). The relative expression level of the target mRNA was normalized to the internal reference GAPDH and calculated by using the 2^−ΔΔCt^ method.

## Western blot

Protein extracted with 1× cell lysis buffer (Promega, USA) was quantified using the BCA Protein Assay Kit (Pierce Biotechnology, Rockford, IL, USA). Proteins (20 µg) in each group were separated by 10% SDS-PAGE (Invitrogen) and then transferred onto PVDF membranes. After that, membranes were blocked in TBST containing 5% skim milk and then incubated at 4°C overnight with primary antibodies purchased from Proteintech (Rosemont, IL, USA), including anti-NAT10 and the loading control anti-GAPDH that were diluted at a ratio of 1: 1000. After washing, membranes were further incubated with the secondary antibody (1: 2000, Proteintech) at room temperature for 2 h. Finally, an enhanced chemiluminescence (ECL) system was used for the visualization of blots.

## Cell counting Kit-8 (CCK-8) assay

CCK-8 kit (Dojindo, Kumamoto, Japan) was used for cell proliferation detection. Briefly, LSCC cells were seeded into 96-well plates at a cell density of 3 × 10^3^ per well. Each well was added with 10 μl CCK-8 reagent and incubated for 2 h. Cell viability was measured at 0, 24, 48, and 96 h after cell attachment by examining the absorbance at 450 nm with a microplate reader.

## Transwell assays

AMC-HN-8 and TU686 cells were inoculated on 24-well transwell chambers (Corning Inc, NY, USA) coated with or without Matrigel at a density of 5 × 10^4^ cell/well for invasion or migration detection. Briefly, the

inner chamber was added with 100 μL serum-free medium while the outer chamber was added with 600 μL medium containing 30% FBS. Twenty-four hours later, cells that migrated or invaded into the outer chamber were fixed with 4% formaldehyde and subjected to crystal violet (0.1%) staining. Five random fields per treatment group were observed using an inverted microscope (Leica, Wetzlar, Germany) for quantification.

## ac4C dot blot

Total RNA was heated to 75°C for 5 min, placed on ice for 1 min, and loaded onto Hybond-N+ membranes. Next, membranes were subjected to crosslink with 150 mJ/cm^2^ by using Stratalinker 2400 (Stratagene, NY, USA) at UV254 nm. After blocking in 0.1% Tween 20 PBS (PBST) containing 5% nonfat milk, the membranes were incubated with the anti-ac4C antibody (Abcam, CA, USA) at 4°C overnight. After washing, the membranes were further incubated with an HRP-conjugated secondary antibody at room temperature for 1 h. Finally, dot blots were visualized by using a chemiluminescent HRP substrate (Millipore, Billerica, MA, USA).

## acRIP-qPCR and NAT10-RIP-qPCR

RIP assays were performed by using an RNA Immunoprecipitation Kit (Geneseed, Guangzhou, China). Briefly, cells were washed twice in PBS and then centrifuged at 1,000 × g for 5 min. Next, cells were treated with 1 mL of RIP lysis buffer on ice for 10 min. Subsequently, antibodies, including anti-ac4C (1: 50, Abcam), anti-NAT10 (1: 50, Proteintech), anti-IgG (1:50, Millipore) was mixed with protein A/G beads at 4°C for 2 h. The beads were then incubated with 450 μL of lysis buffer at 4°C for 2 h. After washing the beads, RNA was extracted from the mixture and analyzed by qPCR.

## Luciferase reporter assay

The FOXM1 3’ UTR region containing the ac4C-modified sites (wild type, WT) and the FOXM1 3’ UTR region with the mutated ac4C-modified sites (mutant type, MUT) were separately inserted into the pmirGLO reporter plasmid (Promega, Madison, WI, USA). The luciferase reporter assay was conducted in LSCC cells following the protocol of the Dual-Luciferase reporter kit (Promega). With NAT10 knockdown, the firefly activities of the abovementioned reporter plasmids were measured by normalizing to the internal control Renilla luciferase.

## RNA stability assay

LSCC cells seeded in 12-well plates were treated with 5 μg/mL of actinomycin D (ActD, Sigma-Aldrich) and incubated for 1, 6, 12, 8, and 24 h. Next, total RNA was isolated and detected by RT-qPCR as abovementioned.

### In vivo *experiments*

The animal experiment was conducted under the approval of the guidelines of 机构. Male BALB/c-nu mice aged 4 weeks (*n* = 12) were purchased from Lingchang Biotechnology Co., Ltd. (Shanghai, China). To establish xenograft models, mice were subcutaneously inoculated with 1 × 10^7^ stably transfected AMC-HN-8 cells and divided into the following four groups: sh-NC (*n* = 3), sh-NAT10 (*n* = 3), sh-NAT10+Vector (*n* = 3) and sh-NAT10+FOXM1 (*n* = 3). After a week, data on tumor volume (calculation formula: length × width× width/2) were collected every week. After continuous growth for 28 days, the mice were sacrificed by cervical vertebrae, and the tumors were removed for tissue collection.

Tissue sections were stained by immunohistochemistry (IHC) to reveal ki67 expression by using the anti-ki67 antibody (1: 200, Abcam), as previously described.^25^

## Statistical analysis

All data obtained from three independent experiments were presented as the mean ± standard deviation (SD) and plotted using GraphPad Prism 6.0 software (GraphPad, CA, USA). SPSS 19.0 (IBM, SPSS, NY, USA) was applied to perform statistical analysis by using Student’s t-test or one-way ANOVA to compare differences between two groups or among more than two groups. Data were defined to be statistically significant when *P* < .05.

## Results

### NAT10 expression level and ac4C level are abundant in LSCC

**Figure 1.**
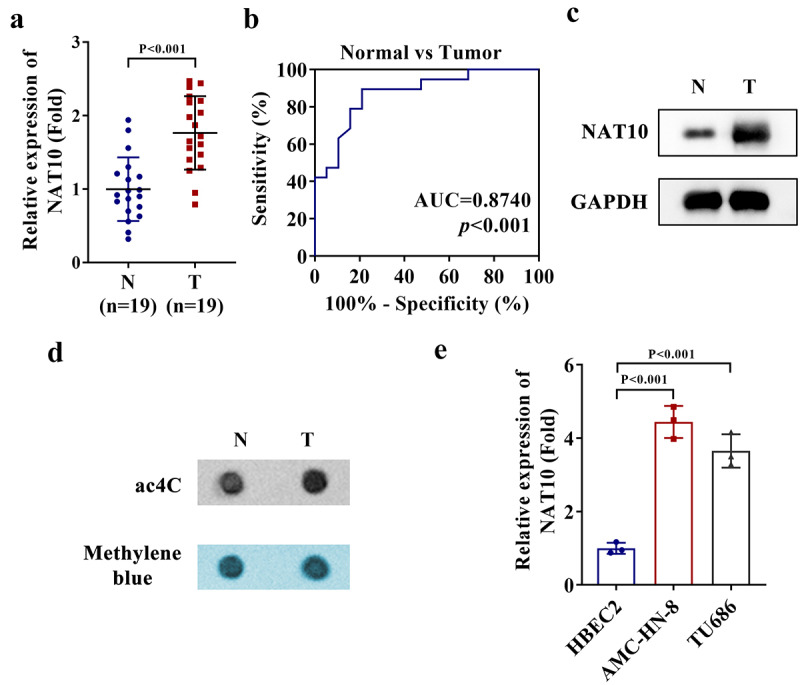
NAT10 expression level and ac4C level are abundant in LSCC.

To clarify the important role of NAT10 in LSCC, we first measured the mRNA level of NAT10 in 19 pairs of LSCC tissues and corresponding adjacent normal tissues through RT-qPCR. The results indicated that the level of NAT10 mRNA was significantly higher in LSCC tissues than in adjacent normal tissues (Figure 1a). Furthermore, we plotted the receiver operating characteristic (ROC) curve and determined that the area under the curve (AUC) value of NAT10 expression (AUC = 0.874) was sufficient for distinguishing the difference between LSCC tissues and adjacent normal tissues (Figure 1b). Moreover, we detected the NAT10 protein level in a pair of tissues using western blot, which showed the high expression of NAT10 in the LSCC tissue (Figure 1c). The pair of tissues were also used for ac4C dot blot, which also showed the high ac4C level in the LSCC tissue (Figure 1d). In addition to detections in tissue samples, we also measured NAT10 expression in LSCC cells via RT-qPCR. Our results indicated a high level of NAT10 mRNA in two LSCC cells (Figure 1e). Therefore, we confirm the abundance of NAT10 expression and ac4C level in LSCC.

a. The mRNA level of NAT10 in 19 pairs of LSCC tissues and corresponding adjacent normal tissues was measured through RT-qPCR (*P* < .001). AU: Please provide a short biography for the all authors as per journal style. b. The AUC curve showing the significance of the NAT10 expression difference between LSCC tissues and adjacent normal tissues (AUC = 0.874, *P* < .001). c. The NAT10 protein level in a pair of LSCC tissue and corresponding normal tissue was measured by western blot. d. The ac4C level in a pair of LSCC tissue and corresponding normal tissue was detected by ac4C dot blot. e. NAT10 expression in two LSCC cells and one normal control cell was measured via RT-qPCR (*P* < .001).

## Knockdown of NAT10 suppresses the proliferation, migration, and invasion of LSCC cells

**Figure 2.**
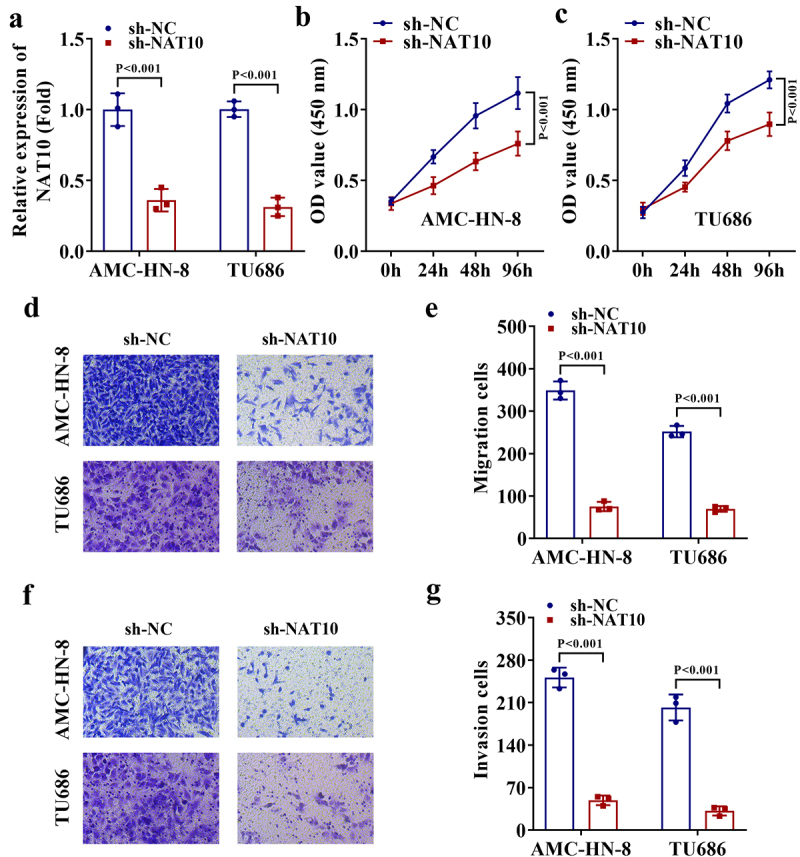
Knockdown of NAT10 suppresses the proliferation, migration, and invasion of LSCC cells.

Considering the high expression of NAT10 in LSCC cells, we established sh-NAT10 and the negative control sh-NC plasmids to stably interfere with NAT10 expression, and the transfection efficiency was verified via qRT-PCR (Figure 2a). Then, CCK-8 assays were performed to evaluate whether NAT10 adjusted the proliferation ability of LSCC cells. According to the results, the proliferation of two LSCC cells was dramatically weakened by the knockdown of NAT10 (Figure 2b–c). Further, we monitored the changes in cell migration and invasion through transwell assays, which showed that NAT10 depletion significantly impaired the abilities of two LSCC cells to migrate and invade (Figure 2d–g). Taken together, NAT10 promotes the proliferation, migration, and invasion of LSCC cells.

a. sh-NAT10 and the negative control sh-NC plasmids were established to stably interfere NAT10 expression in two LSCC cells, and the transfection efficiency was verified via qRT-PCR (*P* < .001). b-c. CCK-8 assays were performed to evaluate the proliferation ability of LSCC cells upon NAT10 knockdown (*P* < .001). d-e. The migration of LSCC cells with NAT10 knockdown was detected by transwell assays (*P* < .001). f-g. The invasion of LSCC cells with NAT10 knockdown was detected by transwell assays (*P* < .001).

## NAT10-mediated ac4C modification stabilizes FOXM1 expression

**Figure 3.**
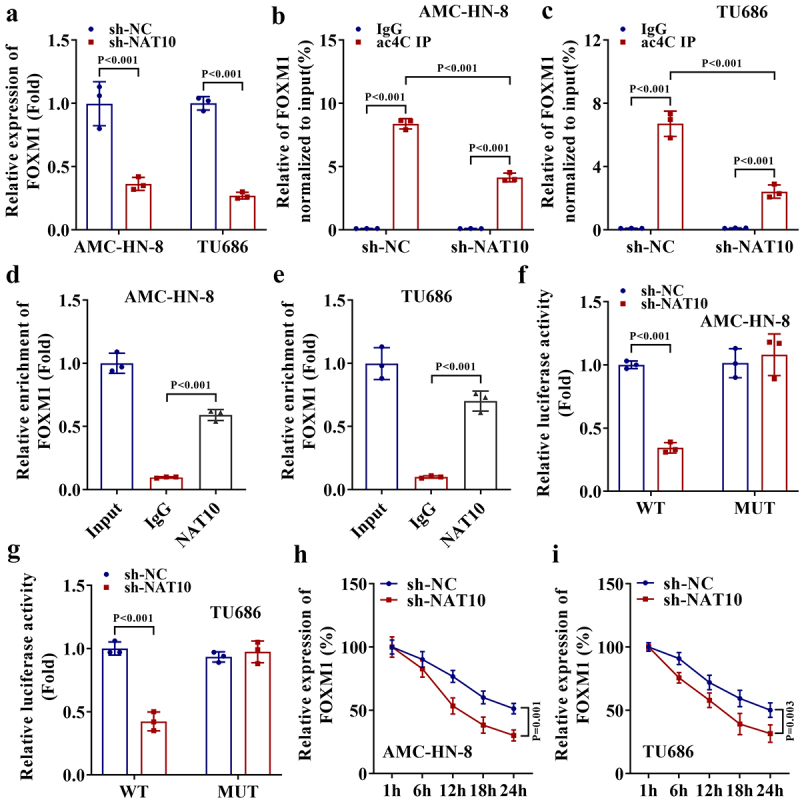
NAT10-mediated ac4C modification stabilizes FOXM1 expression.

FOXM1 is known as a transcription factor promoting the progression of various malignant tumors. Here, we explored the regulating effect of NAT10 on the ac4C level of FOXM1. We first detected the expression level of FOXM1 mRNA upon NAT10 knockdown and determined the downregulation of FOXM1 expression (Figure 3a). Next, we performed acRIP-qPCR and the results indicated that the abundance of ac4C on FOXM1 mRNA could be reduced by NAT10 knockdown (Figure 3b–c). Furthermore, the NAT10-RIP assay demonstrated that NAT10 could interact with FOXM1 mRNA (Figure 3d–e). Then, to verify whether the regulating effect of NAT10 on FOXM1 was attributed to its binding to the FOXM1 3’UTR, we conducted a luciferase reporter assay by establishing the reporter vector containing FOXM1 3’UTR with or without ac4C-modified regions (named WT or MUT). According to the data shown in Figure 3f–g, the luciferase activity of the WT reporter was repressed in two LSCC cells with NAT10 knockdown. Conversely, the MUT group didn’t present significant changes in this regard. Finally, we measured the stability of FOXM1 mRNA in NAT10-silenced LSCC cells treated with ActD. As detected by RT-qPCR, the knockdown of NAT10 shortened the half-life time of FOXM1 (Figure 3h–i). These data demonstrate that NAT10 stabilizes FOXM1 mRNA via ac4C modification.

a. The expression level of FOXM1 mRNA upon knockdown was detected by RT-qPCR (*P* < .001). b-c. acRIP-qPCR was performed to measure the abundance of ac4C on FOXM1 mRNA upon NAT10 knockdown (*P* < .001). d-e. NAT10-RIP assay demonstrated the interaction between NAT10 and FOXM1 mRNA (*P* < .001). f-g. Luciferase reporter assay was conducted to measure the luciferase activity of the reporter vector containing FOXM1 3’UTR with or without ac4C-modified regions (named WT or MUT) in NAT10-silenced LSCC cells (*P* < .001). h-i. The stability of FOXM1 mRNA in NAT10-silenced LSCC cells treated with ActD was evaluated by using RT-qPCR (*P* = .001, *P* = .003).

## NAT10 promotes the malignant processes of LSCC cells through FOXM1

**Figure 4.**
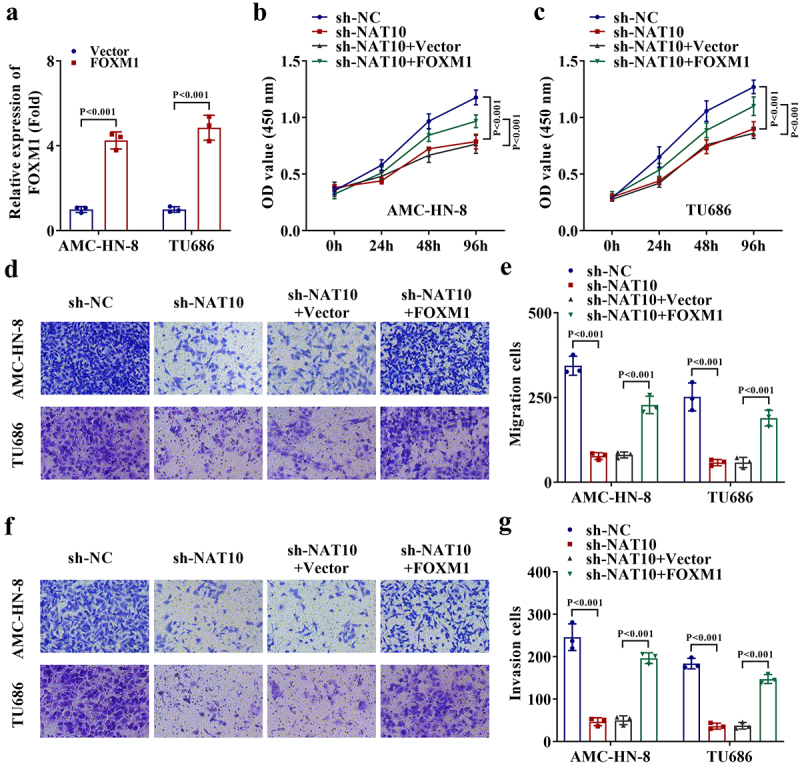
NAT10 promotes the malignant processes of LSCC cells through FOXM1.

For verification of the effects of NAT10-mediated FOXM1 expression changes on LSCC cell functions, rescue assays were performed. Before that, FOXM1 was overexpressed in two LSCC cells (Figure 4a). In subsequent CCK-8 assays, we determined that the ability of LSCC cell proliferation weakened by NAT10 knockdown was recovered by the overexpression of FOXM1 (Figure 4b–c). Additionally, the migrating and invading abilities of LSCC cells suppressed by NAT10 knockdown were rescued by FOXM1 overexpression (Figure 4d–g). Collectively, NAT10 can regulate the proliferation, migration, and invasion of LSCC cells through FOXM1.

a. The transfection efficiency of FOXM1 overexpression vector in two LSCC cells was assessed by RT-qPCR (*P* < .001). b-c. The effect of FOXM1 overexpression on the proliferation ability of NAT10-silenced LSCC cell was measured by CCK-8 assays (*P* < .001). d-e. The effect of FOXM1 overexpression on the migrating ability of NAT10-silenced LSCC cell was assessed by transwell assays (*P* < .001). f-g. The effect of FOXM1 overexpression on the invading ability of NAT10-silenced LSCC cell was assessed by transwell assays (*P* < .001).

## NAT10-mediated FOXM1 upregulation promotes *in vivo* tumorigenesis of LSCC cells

**Figure 5.**
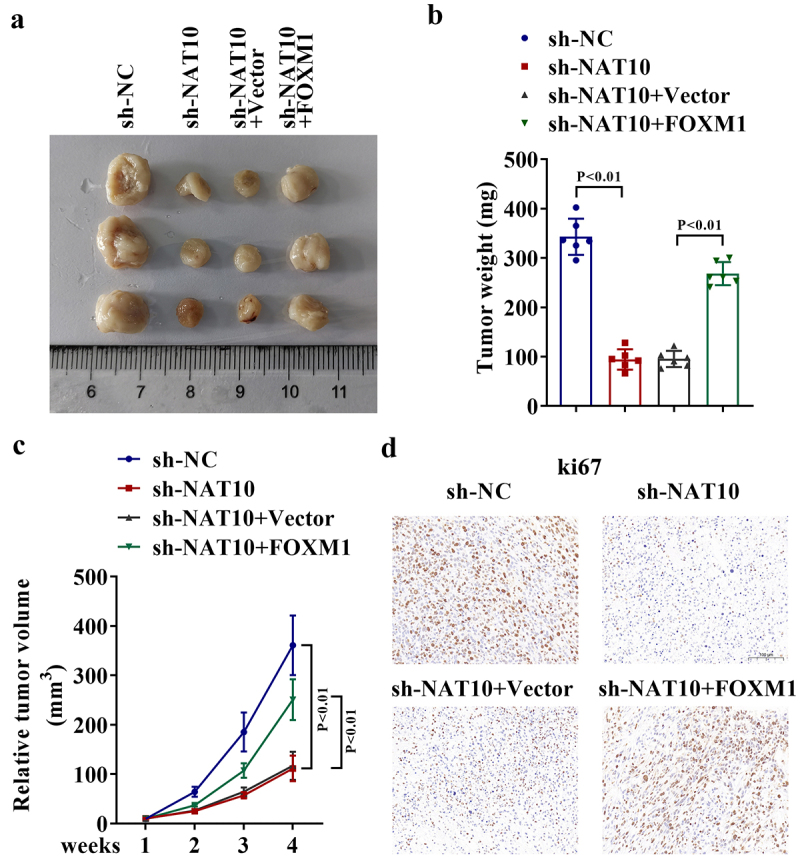
NAT10-mediated FOXM1 upregulation promotes *in vivo* tumorigenesis of LSCC cells.

To further validate the role of NAT10-mediated FOXM1 in LSCC tumor growth, *in vivo* xenograft tumor models were established. AMC-HN-8 cells stably transfected with sh-NC, sh-NAT10, sh-NAT10+Vector, and sh-NAT10+FOXM1 were subcutaneously injected into four groups of nude mice. Four weeks later, each tumor was resected and recorded (Figure 5a). Thereafter, the changes in tumor weight and volume were analyzed and the results revealed that NAT10 silencing inhibited *in vivo* tumor growth, while this tendency was reversed by FOXM1 overexpression (Figure 5b–c). In addition, IHC analysis of tumor tissues in four groups showed that silencing of NAT10 led to the downregulated ki67 expression, while the ki67 expression was enhanced again after overexpression of FOXM1 (Figure 5d). Thus, NAT10 can promote *in vivo* tumorigenesis of LSCC cells through upregulating FOXM1.

a. AMC-HN-8 cells stably transfected with sh-NC, sh-NAT10, sh-NAT10+Vector, sh-NAT10+FOXM1 were subcutaneously injected into four groups of nude mice to establish *in vivo* xenograft tumor models. Four weeks later, each tumor was resected and shown. b-c. The changes in tumor weight and volume were analyzed (*P* < .01). d. IHC analysis of tumor tissues in four groups showed the changes in ki67 expression.

## Discussion

Like most other types of malignant tumors, LSCC accounts for high mortality worldwide, despite the improvement of therapeutic approaches.^26^ To discover new treatment strategies, an in-depth understanding of molecular mechanisms affecting LSCC progression is necessary. NAT10, an acetylation modulator, has been proven to be a cancer-promoting gene by increasing the ac4C level. In our current study, we first measured the mRNA level of NAT10 in tumor and adjacent normal tissues obtained from LSCC patients through surgical resection. We determined that the level of NAT10 mRNA was significantly higher in LSCC tissues. Consistently, the ac4C level was also higher in LSCC tumor tissues. Therefore, we hypothesized that NAT10-mediated ac4C modification might be involved in LSCC progression. Reports have elucidated the importance of NAT10 in tumor progression. For example, NAT10-mediated ac4C modification promotes bladder cancer progression^27^ and drives chemoresistance of bladder cancer cells;^28^ NAT10 accelerates tumor metastasis in gastric cancer via mediating ac4C modification of COL5A1.^29^ Importantly, the potential diagnostic and prognostic role of NAT10 has been revealed in LSCC.^30,31^ To analyze the functional effects of NAT10 on LSCC cells, we established sh-NAT10 and the negative control sh-NC plasmids to stably interfere with NAT10 expression for loss-of-function assays. Through CCK-8 detection, we identified that the proliferation of two LSCC cells was dramatically weakened by the knockdown of NAT10. Further, we monitored the changes in cell migration and invasion through transwell assays, which indicated that NAT10 knockdown efficiently impaired the abilities of two LSCC cells to migrate and invade. Hence, we confirm the promoting role of NAT10 in the proliferation, migration, and invasion of LSCC cells.

Research shows that FOXM1 can exert cancer-promoting functions. For instance, FOXM1 is a prognostic factor and an enhancing factor in small cell lung cancer;^32^ it can enhance the 5-FU resistance of colorectal cancer^33^ and accelerate ovarian cancer cell migration;^34^ FOXM1-mediated molecular pathway can aggravate bladder cancer via modulation of cell cycle process.^35^ Additionally, the critical role of FOXM1 in LSCC has been reported as well.^22–24^ However, whether NAT10-mediated regulating mechanism can alter FOXM1 expression remains unknown. As such, we made mechanism investigations. We first determined that the expression level of FOXM1 mRNA was downregulated upon NAT10 knockdown. Next, we performed acRIP-qPCR and NAT10-RIP assay. Both results indicated that NAT10 could interact with FOXM1 mRNA to affect the abundance of ac4C on FOXM1 mRNA. As reported, ac4C catalyzed by NAT10 on mRNA can increase mRNA stability.^36^ Hence, we performed further luciferase reporter assays and demonstrated that NAT10-mediated regulation on FOXM1 was attributed to the ac4C-modified sites in FOXM1 3’UTR. Finally, we monitored the changes in FOXM1 mRNA stability and found that NAT10 could enhance the stability of FOXM1 mRNA. According to these data, we summarize that NAT10 stabilizes FOXM1 expression via ac4C modification.

To verify the effects of NAT10-mediated FOXM1 expression changes on LSCC progression, we designed and conducted both *in vitro* and *in vivo* rescue assays. Both results indicated that FOXM1 overexpression could counteract the suppressing effects of NAT10 knockdown on the malignant processes and tumorigenesis of LSCC cells.

Collectively, this study demonstrates that NAT10 is upregulated in LSCC and accelerates the malignant processes of LSCC cells. Furthermore, NAT10 can recognize the ac4C sites in FOXM1 3’UTR to stabilize FOXM1 mRNA through ac4C modification. Importantly, FOXM1 can change NAT10-mediated LSCC cell functions. Our findings reveal the tumor-promoting role of NAT10-mediated FOXM1 upregulation in LSCC, which may help to discover novel diagnostic or therapeutic targets for LSCC patients. However, there was still a limitation in this study. In vivo and in vitro data showed that FOXM1 overexpression doesn’t completely compensate for the effect of NAT10 inhibition, which meant that there might be other downstream effectors that can be modified by NAT10 to mediate the development of LSCC. In the future, we will conduct more research to discover more NAT10 modified genes, to improve the specific mechanism of NAT10 in LSCC.

## Data Availability

The datasets used and/or analyzed during the current study are available from the corresponding author upon reasonable request.
